# HLA allelic variation and COVID-19 severity in a Kuwait-based population: a high-resolution immunogenetic study

**DOI:** 10.3389/fimmu.2026.1832089

**Published:** 2026-09-08

**Authors:** Monera Alrukhayes, Nada Al-Shatti, Almunther Alhasawi, Omar Alghubayshi, Fahad Almsned, Mariam M. Al Eissa

**Affiliations:** 1 Department of Medical Laboratory Sciences, Faculty of Allied Health Sciences, Health Sciences Center (HSC), Kuwait University, Shadadiya, Kuwait; 2 Immunology & HLA Laboratory, Kuwait Cancer Control Center, Ministry of Health, Kuwait City, Kuwait; 3 Department of Medicine , Hospital of Infectious Diseases, Jahra, Kuwait; 4 Research Center, King Fahad Specialist Hospital in Dammam (KFSH-D), Dammam, Saudi Arabia; 5 Research Program, Academic, Training, and Research Administration, Eastern Health Cluster, Dammam, Saudi Arabia; 6 School of Systems Biology, George Mason University, Fairfax, VA, United States; 7 Research and Development Department, GeneClinic, Dammam, Saudi Arabia; 8 College of Medicine, AlFaisal University, Riyadh, Saudi Arabia; 9 Population Health Observatory, Ministry of Health, Riyadh, Saudi Arabia; 10 Public Health Authority, Public Health Lab, Riyadh, Saudi Arabia; 11 Research Department, King Khaled Eye Specialist Hospital (KKESH) Research Center, Riyadh, Saudi Arabia; 12 School of Public Policy (KSPP), King Abdullah Petroleum Studies and Research Center (KAPSARC), Riyadh, Saudi Arabia

**Keywords:** COVID-19, HLA, immunogenetics, Kuwait, population genetics, SARS-CoV-2

## Abstract

**Background:**

Human leukocyte antigen (HLA) polymorphisms play a vital role in antiviral immunity, but their influence on COVID-19 severity differs significantly among ethnic groups. T-cell responses are essential for clearing SARS-CoV-2, yet the immunogenetic factors underlying the diverse clinical outcomes in COVID-19, especially in certain populations, are not fully understood. In a Kuwait-based cohort from national referral and public health facilities, we examined HLA allelic variants to identify potential protective or risk factors related to COVID-19 severity.

**Methods:**

We studied 494 participants: 75 healthy controls and 419 COVID-19 patients, classified by severity (Recovered, Moderate, Severe, Critical/ICU). HLA alleles at nine loci (HLA-A, -B, -C, -DRB1, -DRB3/4/5, -DQA1, -DQB1, -DPA1, -DPB1) were genotyped via next-generation sequencing. Associations with severity were tested using logistic regression, adjusting for age, sex, and ABO blood group, with the Severe/ICU group as the reference. This was repeated, excluding controls from the reference, to separate severity effects from susceptibility to infection.

**Results:**

HLA genotype was successfully linked to complete clinical/covariate data for 364 of 494 participants (74%; 60 Healthy, 77 Recovered, 125 Moderate, 66 Severe, 36 ICU), though linkage success was lower among ICU patients (47%) than other strata (75–82%), a difference we consider more likely attributable to attrition during critical care than to allele-related bias. Among these 364 participants, 22 HLA alleles spanning all nine genotyped loci showed nominal association with severe/critical disease (P < 0.10) in the primary model, predominantly risk-conferring: HLA-C*06:02:01 (OR 2.50, 95% CI 1.43–4.36, P = 0.001), HLA-DQB1*02:02:01 (OR 2.25, 1.31–3.88, P = 0.003), HLA-DRB1*15:03:01 (OR 4.65, 1.38–15.67, P = 0.013), HLA-A*29:02:01 (OR 14.11, 1.43–138.81, P = 0.023), and HLA-B*13:02:01 (OR 12.78, 1.31–124.18, P = 0.028), among others; one protective association was identified, HLA-A*31:01:02 (OR 0.26, 0.08–0.83, P = 0.023). These five associations remained directionally concordant in the sensitivity analysis excluding Healthy controls, whereas several weaker associations did not. None survived correction for multiple testing (FDR-adjusted q ranged from 0.36 to 0.99); a minimum-detectable-odds-ratio calculation indicated this cohort was powered to detect only large effects (OR ≈ 10 for a 1%-frequency allele after correction), consistent with underpowering rather than absence of true effect for several low-frequency candidates. Age remained the strongest independent predictor of severity (P < 0.001), whereas sex showed no significant association after adjustment (P = 0.289).

**Conclusion:**

This study provides a comprehensive characterisation of HLA allele frequencies in a Kuwait-based population, including their association with COVID-19 clinical outcomes. Age was a substantially more dominant determinant of COVID-19 severity than HLA genotype in this cohort. None of the identified HLA alleles retained statistical significance after correction for multiple testing; given both this power limitation and the absence of individual-level ancestry and vaccination data in a population with substantial genetic and demographic heterogeneity, these findings should be regarded as hypothesis-generating candidates for replication in larger, ancestry-adjusted, adequately powered cohorts.

## 1 Introduction

In March 2020, the World Health Organisation (WHO) declared coronavirus disease 2019 (COVID-19), caused by severe acute respiratory syndrome coronavirus 2 (SARS-CoV-2), a pandemic. Since the virus emerged in late 2019, it has dramatically reshaped global health systems. SARS-CoV-2 is the seventh known human coronavirus capable of causing significant respiratory illness (1–3). Although the immunological determinants of COVID-19 disease severity and long-term immunity remain incompletely understood, governments and pharmaceutical companies have competed to control this pandemic against SARS-CoV-2 (4). T-cell-mediated immunity, predominantly mediated by cytotoxic CD8 cells, is pivotal to viral clearance. Nevertheless, much remains unknown about the long-term stability, durability, and diversity of these responses across populations (5–7). Nonetheless, COVID-19 severity is shaped by complex interactions among viral factors, host immune genetics, and environmental determinants.

Encoded by the major histocompatibility complex (MHC) on chromosome 6, human leukocyte antigen (HLA) molecules govern adaptive immunity by mediating antigen presentation to T cells (8). These molecules are categorised into two primary classes: Class I (HLA-A, -B, -C), which present intracellular peptides (including tumour and viral fragments) to CD8^1^ cytotoxic T cells, and Class II (HLA-DR, -DP, -DQ), which display exogenous antigens to CD4^1^ helper T cells (9). As the most polymorphic region of the human genome, HLA variation drives significant inter-individual and inter-population heterogeneity in immune responsiveness, directly influencing susceptibility to infectious diseases (10). Distinct HLA alleles possess unique peptide-binding specificities that determine the breadth and magnitude of the antiviral T-cell repertoire (11). This genetic regulation has been highlighted by research on previous outbreaks of infectious disease. For example, during the 2003 SARS outbreak, the HLA-B*46:01 allele was strongly associated with disease severity (12). Similarly, in silico analyses of SARS-CoV-2 have predicted that HLA-B*46:01 exhibits restricted peptide binding, potentially compromising viral control, whereas HLA-B*15:03 is predicted to confer broader, cross-protective immunity (13). To determine whether the results of such computational modelling can be extrapolated to clinical settings, studies in genetically diverse populations are needed. Recent studies of large populations reported that HLA associations with COVID-19 often vary across ethnic groups and may also be affected by socioeconomic or exposure-related variables (14). Moreover, recent evidence has shown that HLA diversity has effects beyond susceptibility to infection, including lymphocyte depletion, inflammatory dysregulation, thrombosis risk, and cytokine-mediated tissue damage (10).

Kuwait’s population includes substantial representation of Middle Eastern, North African, and South Asian ancestries nationally, making it a population of interest for studying viral pathogenicity. HLA-A*02:01, HLA-C*06:02, and HLA-B*50:01 are the predominant Class I alleles among Kuwaitis, while HLA-DQB1*02:01 and HLA-DRB1*07:01 dominate the Class II repertoire, as established by a pioneering study of 595 individuals (15). To develop effective vaccines and identify cross-reactivity against emerging coronaviruses, it is essential to characterise the population-specific HLA landscape in Kuwait and its associations with disease severity. The resolution of HLA genotyping also has a major impact on downstream associations. Low-resolution HLA typing methods can yield ambiguous results about which alleles bind and present specific antigenic peptides, a problem sometimes resolved only by more advanced high-resolution next-generation sequencing platforms (9). To overcome the limitations of previous work and strengthen the evidence for HLA–COVID-19 associations, high-resolution techniques should be used, with adjustments for parameters such as demographics and clinical confounders, to ensure that immunogenetic effects are accurately determined (14). In this study, by integrating high-resolution HLA genotyping with comprehensive clinical stratification, we aimed to characterise the immunogenetic landscape associated with COVID-19 severity in a Kuwait-based population of nationals and residents. We had the following specific aims (1): To measure HLA allele frequencies in Kuwait-based individuals across nine HLA loci spanning the full clinical spectrum of COVID-19 infection as well as in uninfected individuals; and (2) To characterise HLA alleles that retain a statistically significant association with disease severity after adjustment for age, sex, and ABO blood group. This work should serve as a valuable reference for immunogenetic risk stratification. It is hoped that this study will inform efforts to develop effective public health strategies against emerging coronaviruses and other viral diseases, as well as to develop vaccines tailored to local populations.

This work characterises HLA allele frequencies and their links to COVID-19 severity in Kuwait, creating a reference for immunogenetic research in an underrepresented population. Such data are vital for future studies guiding public health against coronaviruses and other viruses.

## 2 Materials and methods

### 2.1 Ethical approval

This study was reviewed and approved by the Ethics Committee for the Protection of Human Subjects at the Kuwait Ministry of Health (Protocol No. 1525/2020). Written informed consent was obtained from all participants and from the parents and guardians of those under 18 prior to enrolment. All clinical and genetic data were pseudo-anonymised to protect participant confidentiality, in accordance with the Declaration of Helsinki.

### 2.2 Participants and recruitment

A total of 625 participants (aged 11–96 years) were recruited between December 2020 and July 2021: 548 COVID-19 patients from Jaber Al-Ahmad Al-Jaber Al-Sabah Hospital Kuwait’s primary COVID-19 referral centre and designated polyclinics, and 77 healthy controls from the Public Health Headquarters. SARS-CoV-2 infection was confirmed by RT-PCR of nasopharyngeal swabs; healthy controls had no history of COVID-19 and were seronegative on the LIAISON SARS-CoV-2 S1/S2 IgG assay. However, because this assay cannot distinguish vaccine- from infection-induced anti-spike antibodies, it may have inadvertently selected for unvaccinated controls (see Limitations).

Clinical severity among COVID-19 patients was stratified according to U.S. FDA case severity definitions (**Supplementary Table 1**) into Mild (fever, cough, sore throat, malaise, headache, myalgia, nausea, vomiting, diarrhoea, or loss of taste/smell, without dyspnoea or signs of higher severity), Moderate (mild symptoms or exertional dyspnoea; respiratory rate ≥20/min, heart rate ≥90/min, SpO_2_ >93% on room air at sea level), Severe (respiratory rate ≥30/min, heart rate ≥125/min, SpO_2_ ≤93% on room air, or PaO_2_/FiO_2_ <300), and Critical/ICU (respiratory failure requiring advanced respiratory support, shock, or multi-organ dysfunction/failure). Cases originally coded “Minor” at data entry (n = 104), corresponding to the Mild category above, were harmonised into Moderate prior to analysis. The Recovered group comprised individuals enrolled within 90 days of confirmed infection with fully resolved symptoms.

Pre-specified exclusion criteria active immunosuppression, malignancy, or transplant status; co-existing confirmed respiratory infection at admission; documented autoimmune disease with known HLA associations; failed or ambiguous HLA genotyping at more than one locus; incomplete clinical documentation; and, among controls, detectable SARS-CoV-2 IgG antibodies — excluded 131 participants (129 patients, 2 controls), yielding a final analytical sample of 494 participants (419 COVID-19 patients, 75 healthy controls) with complete medical records (**Table 1**). Within the retained cohort, 12 participants had missing age data, and 7 had missing sex data, primarily reflecting operational constraints during peak pandemic-surge admissions; these were excluded listwise only from the corresponding age- or sex-adjusted regression models and retained in all other analyses.

**Table 1:** Baseline demographic and clinical characteristics of the study cohort (n=494), stratified by group.

Characteristic	N	COVID-19 Patients N = 419	Healthy Controls N = 75
Age (years)	482	58.3 ± 15.8; 59.0 (48.0–70.0); 11.0–96.0	43.5 ± 12.5; 42.0 (35.0–52.0); 22.0–76.0
Missing		11	1
Sex	487		
Female		212.0 (51.5%)	40.0 (53.3%)
Male		200.0 (48.5%)	35.0 (46.7%)
Missing		7	0
ABO group	472		
A		108.0 (27.1%)	28.0 (37.8%)
B		114.0 (28.6%)	16.0 (21.6%)
O		176.0 (44.2%)	30.0 (40.5%)
Missing		21	1
Diagnosis	494		
Healthy		0.0 (0.0%)	75.0 (100.0%)
ICU		77.0 (18.4%)	0.0 (0.0%)
Moderate		153.0 (36.5%)	0.0 (0.0%)
Recovered		101.0 (24.1%)	0.0 (0.0%)
Severe		88.0 (21.0%)	0.0 (0.0%)

Data are presented as mean ± SD, median (Q1–Q3), and min–max for continuous variables, and as n (%) for categorical variables. The cohort comprises COVID-19 patients (n = 419), classified by clinical severity into Recovered, Moderate, Severe, and Critical/ICU categories according to U.S. FDA severity case definitions (**Supplementary Table 1**), and Healthy Controls (n = 75). Missing values are reported per variable: age (n = 12), sex (n = 7), and ABO blood group, attributable to incomplete intake documentation, primarily during emergency-level admissions at peak pandemic surges.

Of these 494 participants, HLA genotype was successfully linked to clinical records for 364 (60 Healthy, 77 Recovered, 125 Moderate, 66 Severe, 36 ICU); the remaining 130 had clinical records but no corresponding genotyping entry, most plausibly reflecting sample volume, collection timing, or sequencing-stage attrition not systematically recorded for this cohort. This attrition was not uniform across severity strata: linkage succeeded for only 47% of ICU patients (36 of 77), compared with higher, more consistent rates across the Healthy, Recovered, Moderate, and Severe strata. Descriptive demographic and clinical analyses (Table 1–3) draw on the full cohort of 494 participants; HLA association testing (Tables 5–6) is restricted to the 364 participants with linked genotype and clinical data.

### 2.3 Sample collection, DNA extraction, and HLA genotyping

Peripheral blood samples (2–4 mL) were collected in EDTA tubes during routine venipuncture. Genomic DNA was extracted from whole blood, buffy coats, or peripheral blood mononuclear cells (PBMCs) using the Qiagen DNeasy Blood & Tissue Kit (Qiagen, Germantown, MD, USA) according to the manufacturer’s instructions. High-resolution genotyping of nine HLA loci (HLA-A, -B, -C, -DRB1, -DRB3/4/5, -DQA1, -DQB1, -DPA1, -DPB1) was performed by next-generation sequencing. Libraries were prepared using ScisGo™ HLA v3.0 typing kits (Scisco Genetics Inc., Seattle, WA, USA), which involved amplicon PCR, enzymatic cleanup, barcoding, and magnetic bead-based size selection. Sequencing was performed on the Illumina MiSeq platform. Alleles were called to 3-field (6-digit) resolution using the manufacturer’s bioinformatics pipeline, with ambiguous calls resolved to 2-field resolution.

### 2.4 Statistical analysis

All statistical analyses were conducted in R (version 4.6.1; R Foundation for Statistical Computing, Vienna, Austria) via RStudio (2026.07.1, Build 147). Data import, cleaning, and transformation used readr (v2.2.0), dplyr (v1.2.1), tidyr (v1.3.2), tibble (v3.3.1), stringr (v1.6.0), and janitor (v2.2.1); descriptive and analytical summary tables were generated with gtsummary (v2.5.1) and exported using flextable (v0.10.0) and officer (v0.7.6); graphics were produced with ggplot2 (v4.0.3), with workflow support from upstartr (v0.2.0). Continuous variables were summarised as mean ± SD or median (IQR) as appropriate, and categorical variables as frequencies and percentages.

Baseline characteristics across the five study groups Healthy, Recovered, Moderate, Severe, and Critical/ICU were compared on the full clinical cohort of 494 participants (75 Healthy, 77 ICU, 153 Moderate, 101 Recovered, 88 Severe; **Table 1**) using the Kruskal–Wallis rank-sum test for continuous variables and Pearson’s chi-square or Fisher’s exact test for categorical variables, depending on expected cell counts.

Three complementary regression models, each fitted to this same 494-participant cohort and each adjusted for age (continuous, per 1-year increment) and sex (female as reference), addressed distinct inferential questions about the relationship between age, sex, and severity. A binary logistic regression (epitools v0.5.10.1) estimated the odds of hospitalisation (Moderate/Severe/Critical-ICU vs. Healthy/Recovered), yielding odds ratios with 95% confidence intervals. A multinomial logistic regression (nnet v7.3.20), with Healthy as the reference category, estimated relative risk ratios for each severity stratum individually, preserving the contrast between each disease state and the unaffected population. An ordinal proportional-odds model (MASS v7.3-66) tested whether age predicted position along the ordered continuum of confirmed disease states (Recovered → Moderate → Severe → Critical/ICU); Healthy controls were excluded from this model because the Healthy|Recovered boundary reflects a sampling-design contrast — deliberately recruited younger, uninfected individuals versus confirmed COVID-19 patients — rather than a step on a clinical severity continuum, with that age–hospitalisation contrast instead captured by the binary and multinomial models above. Participants with missing age or sex were excluded from all three models on a complete-case basis. The proportional-odds assumption was formally verified using the Brant test (omnibus χ² = 2.37, df = 3, P = 0.50), and goodness-of-fit for the binary logistic model was assessed using Nagelkerke R² (0.268).

For HLA-based analyses, raw allele calls were linked to participant identifiers and merged with demographic and clinical metadata (age, sex, ABO blood group, clinical diagnosis). This complete-case restriction yielded an analytic sample of 364 participants (60 Healthy, 77 Recovered, 125 Moderate, 66 Severe, 36 ICU), used uniformly for genotype call-rate assessment, the missingness-versus-outcome test, allele frequency estimation and its corresponding figure, and all covariate-adjusted association testing. Attrition from the full clinical cohort to this analytic sample was not uniform across strata: the ICU group showed the largest proportional reduction (77 to 36 participants, 53%), compared with 18–25% reductions elsewhere.

Within this 364-participant sample, HLA genotype calls were curated descriptively before inferential testing: for each locus, the two alleles carried by each participant were expanded into long format, duplicate allele entries per participant were collapsed, and allele frequencies were calculated by diagnostic group and locus as the count and percentage of participants carrying each allele relative to the total allele count (2N) in that group and locus, with 95% Wilson confidence intervals; the five most frequent alleles per locus and group were ranked and tabulated. These descriptive frequencies characterised the cohort’s baseline allelic landscape and informed the alleles carried forward into association testing.

For association testing, the sample was dichotomised into a Severe Outcome group (Severe or ICU) and a Non-Severe Reference group, with two reference-group definitions analysed in parallel: a primary model (reference = Moderate, Recovered, and Healthy) and a sensitivity model excluding Healthy controls from the reference (Moderate and Recovered only), isolating severity-specific effects from susceptibility-to-infection effects. HLA–disease associations were tested using hlaAssocTest in the HIBAG R/Bioconductor package (v1.48.1; Bioconductor 3.19), fitted separately for each locus to avoid pseudoreplication from pooling loci into a single long-format dataset: within each locus, the 1% phenotypic-frequency threshold was re-derived, a locus-specific hlaAllele genotype object was constructed, and a dominant-model generalised linear model (allele carriers, heterozygous or homozygous, versus non-carriers) with a logit link was fitted, adjusted for age, sex, and ABO blood group. Per-locus results were pooled across all loci, and a single Benjamini–Hochberg false discovery rate correction was applied to the complete pooled set of 279 tested alleles (verified against the confirmed 364-participant sample), preserving the correct multiple-testing burden. Concordance between the primary and sensitivity models was assessed by comparing the nominal significance (P < 0.05) for each allele across both models. Alleles with P < 0.10 in the primary model are reported in the main-text significant-associations table; full results for all tested alleles, including exact p-values and FDR-adjusted p-values, are provided in the **Supplementary Tables**.

## 3 Results

### 3.1 Baseline characteristics of the study cohort

Immunogenetic and clinical data were obtained from 75 healthy controls and 419 COVID-19 patients (N = 494; **Table 1**). Healthy controls were, on average, younger than patients: mean age ± SD was 43.5 ± 12.5 years (median 42.0, IQR 35.0–52.0, range 22.0–76.0) among controls, compared with 58.3 ± 15.8 years (median 59.0, IQR 48.0–70.0, range 11.0–96.0) among patients (age data missing for 11 patients and 1 control; N = 482). Sex distribution was comparable between groups: 212 (51.5%) females and 200 (48.5%) males among patients, and 40 (53.3%) females and 35 (46.7%) males among healthy controls (sex data missing for 7 patients; N = 487).

ABO blood group distribution was similar across groups (N = 472; missing data for 21 patients and 1 control): among patients, group O was most common (176, 44.2%), followed by group B (114, 28.6%) and group A (108, 27.1%); among healthy controls, group O was also most common (30, 40.5%), followed by group A (28, 37.8%) and group B (16, 21.6%). Among the 419 COVID-19 patients, clinical severity was as follows: 153 (36.5%) Moderate, 101 (24.1%) Recovered, 88 (21.0%) Severe, and 77 (18.4%) Critical/ICU.

Sex distribution did not differ significantly across the five clinical severity strata (75 [15%] Healthy, 100 [21%] Recovered, 151 [31%] Moderate, 88 [18%] Severe, 73 [15%] ICU; Pearson’s χ² test, P = 0.8). Females and males showed nearly identical distributions across categories (e.g., 30% vs 32% Moderate, 17% vs 19% Severe, 17% vs 13% ICU), indicating no evidence of a sex-based skew in disease severity in this cohort (**Table 2A**). Hospitalisation rates were virtually identical between sexes, with 64% of females (161/252) and 64% of males (151/235) requiring hospitalisation (Fisher’s exact test, P > 0.9). This finding indicates no evidence of a sex-based difference in the likelihood of hospitalisation in this cohort (**Table 2B**). In age-adjusted logistic regression, male sex was not significantly associated with hospitalisation (OR 1.26, 95% CI 0.83–1.92, P = 0.3), whereas age showed a strong, independent association, with each additional year increasing the odds of hospitalisation by 7% (OR 1.07, 95% CI 1.06–1.09, P < 0.001) (**Table 2C**). Multinomial logistic regression, using the Healthy group as reference, confirmed a graded increase in age-related risk across the severity spectrum: each additional year of age increased the relative risk of belonging to the Recovered group by 3% (RRR 1.03, 95% CI 1.00–1.05, P = 0.025), Moderate by 7% (RRR 1.07, 95% CI 1.04–1.09, P < 0.001), Severe by 11% (RRR 1.11, 95% CI 1.08–1.14, P < 0.001), and ICU by 13% (RRR 1.13, 95% CI 1.10–1.16, P < 0.001). This progressive gradient mirrors the ordinal severity scale and supports age as a dose-dependent risk factor. Sex showed no significant association with any stratum relative to Healthy (RRR range 1.10–1.51, all P > 0.2), consistent with the null sex effect in the binary hospitalisation model (OR 1.26, 95% CI 0.83–1.92, P = 0.289), confirming this finding holds across the full clinical spectrum (**Table 2D**).

**Table 2A:** Distribution of male and female participants across the five clinical severity strata (healthy, recovered, moderate, severe, ICU).

	Diagnostic groups						
Sex	Healthy	Recovered	Moderate	Severe	ICU	Total	p-value^1^
Female	40 (16%)	51 (20%)	76 (30%)	43 (17%)	42 (17%)	252 (100%)	
Male	35 (15%)	49 (21%)	75 (32%)	45 (19%)	31 (13%)	235 (100%)	0.8
Total	75 (15%)	100 (21%)	151 (31%)	88 (18%)	73 (15%)	487 (100%)	

Data are presented as n (%) by sex. Differences in sex distribution across strata were assessed using Pearson’s chi-square test (χ² test, P = 0.8); no significant sex-based skew in disease severity was observed in this cohort.

^1^ Pearson’s chi-square test.

^2^ Row % and p-value from chi-square test; Fisher’s exact test used if expected counts < 5.

**Table 2B:** Comparison of hospitalisation rates (hospitalised vs. non-hospitalised) between male and female participants.

	Hospitalisation status			
Sex	Non-hospitalised	Hospitalised	Total	p-value^1^
Female	91 (36%)	161 (64%)	252 (100%)	
Male	84 (36%)	151 (64%)	235 (100%)	>0.9
Total	175 (36%)	312 (64%)	487 (100%)	

Data are presented as n (%) by sex. Hospitalisation was defined as meeting criteria for Moderate, Severe, or Critical/ICU disease; non-hospitalised comprised Healthy and Recovered individuals. The P-value was calculated using Fisher’s exact test (P > 0.9); no significant sex-based difference in the likelihood of hospitalisation was observed in this cohort.

^1^ Fisher’s exact test.

^2^ Row % and p-value from Fisher’s exact test (2×2).

**Table 2C:** Odds ratios (ORs) and 95% confidence intervals (CIs) for the associations of sex and age with the likelihood of hospitalisation.

Characteristic	OR^1^	95% CI	p-value
Sex (male vs. female)			0.3
Female	—	—	
Male	1.26	0.83, 1.92	
Age (years)	1.07	1.06, 1.09	<0.001

Male sex was not significantly associated with hospitalisation (OR 1.26, 95% CI 0.83–1.92, P = 0.289). Each additional year of age increased the odds of hospitalisation by approximately 7% (OR 1.07, 95% CI 1.06–1.09, P < 0.001).

CI = confidence interval, OR = odds ratio^1^.

^1^ Binary logistic regression with hospitalisation (Moderate/Severe/Critical-ICU vs. Healthy/Recovered) as the outcome, adjusted for age (continuous, per 1-year increment) and sex (female as reference). Model fit: Nagelkerke R² = 0.268, Cox & Snell R² = 0.195, McFadden R² = 0.167.

**Table 2D:** Multinomial and binary logistic regression models evaluating age and sex as predictors of COVID-19 severity.

Model^1^	Outcome	Parameter	Effect	Estimate (95% CI)	p-value
Logistic	Hospitalised	(Intercept)	OR	0.04 (0.02–0.09)	<0.001
		Age (years)	OR	1.07 (1.06–1.09)	<0.001
		Sex (male vs. female)	OR	1.26 (0.83–1.92)	0.289
Multinomial (Diagnostic group)	ICU	(Intercept)	RRR	0.00 (0.00–0.01)	<0.001
		Age (years)	RRR	1.13 (1.10–1.16)	<0.001
		Sex (male vs. female)	RRR	1.10 (0.53–2.26)	0.804
	Moderate	(Intercept)	RRR	0.07 (0.02–0.22)	<0.001
		Age (years)	RRR	1.07 (1.04–1.09)	<0.001
		Sex (male vs. female)	RRR	1.39 (0.77–2.52)	0.273
	Recovered	(Intercept)	RRR	0.39 (0.13–1.19)	0.097
		Age (years)	RRR	1.03 (1.00–1.05)	0.025
		Sex (male vs. female)	RRR	1.15 (0.62–2.14)	0.648
	Severe	(Intercept)	RRR	0.00 (0.00–0.01)	<0.001
		Age (years)	RRR	1.11 (1.08–1.14)	<0.001
		Sex (male vs. female)	RRR	1.51 (0.76–3.00)	0.235

Odds ratios (OR) are reported for the binary hospitalisation model, and relative risk ratios (RRR) are reported for the multinomial model comparing each severity stratum with the Healthy reference group.

OR, odds ratio; RRR, relative risk ratio.

^1^ Binary logistic model: hospitalisation (Moderate/Severe/Critical-ICU vs. Healthy/Recovered) as outcome. Multinomial model: ‘Healthy’ as reference category, estimating the relative risk of belonging to each severity stratum (Recovered, Moderate, Severe, ICU). Both models were adjusted for age (continuous, per 1-year increment) and sex (female as reference); results are presented as OR/RRR with 95% CI and two-sided P-value. The multinomial model addresses stratum-specific contrasts that binary or ordinal models cannot capture.

To characterise the relationship between age and disease severity among confirmed COVID-19 patients, the ordinal proportional-odds model was re-specified to include only the four ordered COVID-19 severity strata (Recovered, Moderate, Severe, Critical/ICU), excluding Healthy controls, because the original Healthy|Recovered threshold exhibited near-perfect empirical separation reflecting a sampling-design contrast rather than a substantive severity gradient. In this revised model, age remained significantly associated with higher clinical severity (**Table 3A**): each additional year of age increased the odds of being in a higher-severity category by approximately 5.6% (OR 1.056, 95% CI 1.043–1.069, P < 0.001) (**Table 3B**). Model fit improved relative to the original five-category specification (AIC 1024.6, deviance 1016.6), consistent with removal of the separation-affected boundary. However, the Recovered|Moderate threshold in this revised model retained an anomalously small standard error (intercept 1.844, SE 0.006, z = 292.02) relative to the Moderate|Severe (intercept 3.719, SE 0.362, z = 10.27) and Severe|Critical-ICU (intercept 4.945, SE 0.399, z = 12.40) thresholds (**Table 3C**), indicating that near-complete separation persists at this boundary. This is consistent with the Recovered stratum’s sampling definition (individuals recruited within 90 days of confirmed infection with fully resolved symptoms), which likely represents a distinct, younger sampling frame rather than a point on the same severity continuum as Moderate, Severe, and Critical/ICU. Accordingly, we present the age coefficient from this model as indicative rather than definitive, and interpret it alongside the binary logistic and multinomial models (Table 2), which are not affected by this artefact and provide the primary basis for our conclusion that age is strongly associated with COVID-19 severity in this cohort.

**Table 3A:** Odds ratio for age from the ordinal proportional-odds model predicting COVID-19 severity.

Characteristic	OR	95% CI
Age (years)	1.06	1.04, 1.07

Severity was modelled as an ordered outcome across four strata (Recovered < Moderate < Severe < Critical/ICU), excluding Healthy controls. Results are from a proportional-odds logistic regression (logit link) with age as the sole predictor.

CI, confidence interval, OR, odds ratio.

**Table 3B:** Effect estimate and model fit for the ordinal age–severity model.

Parameter	Estimate	95% CI/Fit	p-value	Interpretation
Age (per year)	OR = 1.056	[1.043, 1.069]	<0.001	5.6% increase in odds per year
Model fit	AIC = 1024.6	Deviance = 1016.6	--	Model comparison metric

Reports the age coefficient (odds ratio, 95% confidence interval, and P-value) from the re-specified four-category proportional-odds model (Recovered, Moderate, Severe, Critical/ICU; Healthy controls excluded), along with model fit statistics (AIC, deviance).

**Table 3C:** Threshold (cutpoint) estimates from the ordinal age–severity model.

Severity Threshold	Intercept	Std. Error	z-value
Recovered| Moderate	1.844	0.006	292.02
Moderate|Severe	3.719	0.362	10.27
Severe|Clinical/ICU	4.945	0.399	12.40

Reports the intercept, standard error, and z-value for each of the three severity-category boundaries in the re-specified four-category proportional-odds model (Recovered, Moderate, Severe, Critical/ICU; Healthy controls excluded).

Genotyping call rates were 100% across all nine HLA loci and all five diagnostic groups (n = 364 participants with complete clinical–genetic data: 60 Healthy, 36 ICU, 125 Moderate, 77 Recovered, and 66 Severe), indicating no locus-specific genotyping failure or differential missingness by disease severity (**Supplementary Table 2**). Because call completeness was uniform across loci, the corresponding missingness-versus-outcome test yielded an identical result at each locus (P = 6.04 × 10^-13^; **Supplementary Table 3**). This uniform result reflects the fixed completeness and diagnostic-group distribution rather than a locus-specific missingness effect, supporting the absence of genotyping-related bias in the downstream association analyses.

**Figure 1:**
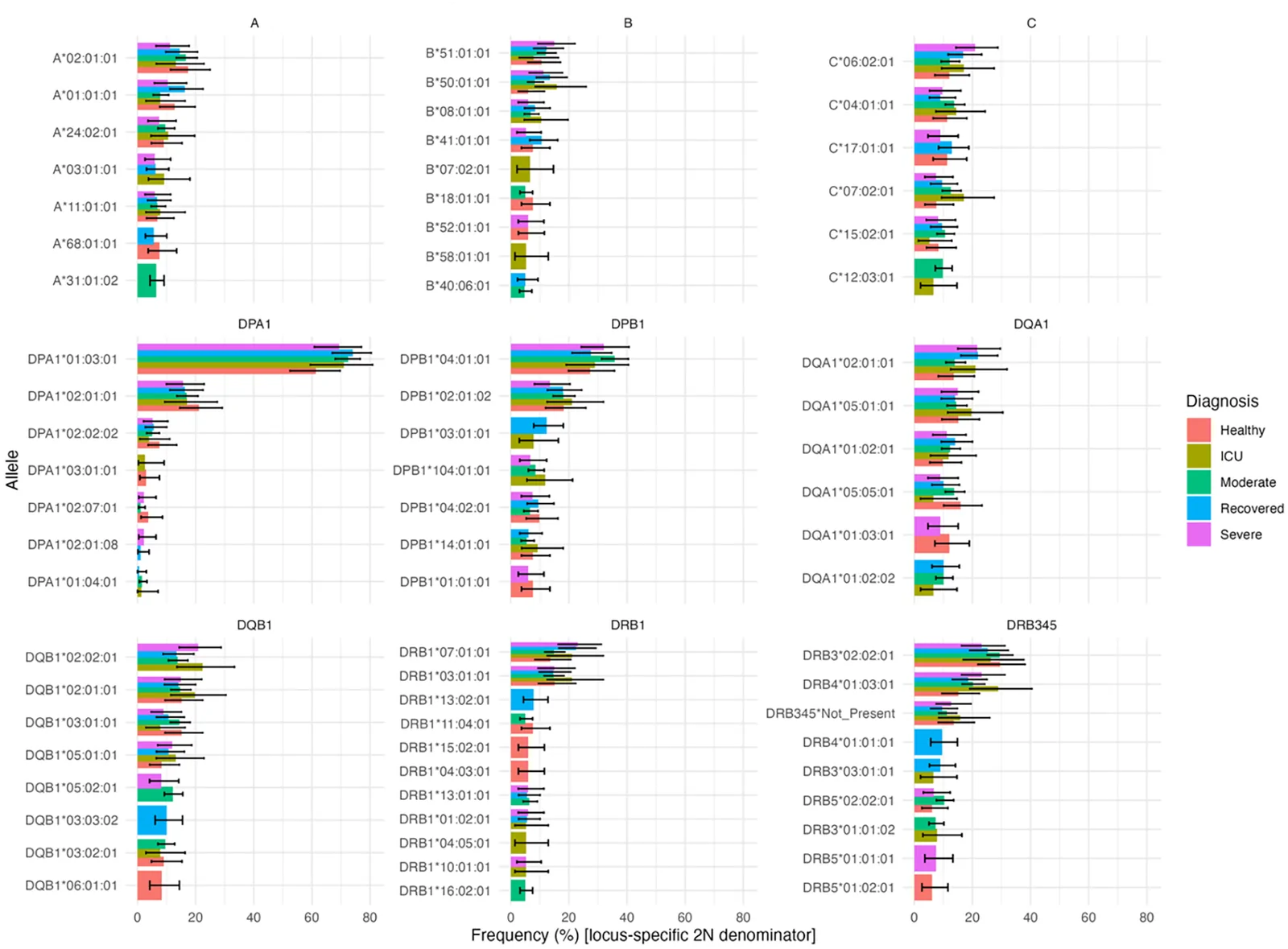
Per-locus HLA allele frequencies by diagnostic group. Frequencies (%) of the most common HLA alleles across the nine genotyped loci (HLA **(A–C)** -DPA1, -DPB1, -DQA1, -DQB1, -DRB1, and -DRB3/4/5), stratified by diagnostic group (Healthy, ICU, Moderate, Recovered, and Severe). Allele frequencies were calculated using a locus- and group-specific 2N denominator, corresponding to twice the number of participants contributing genotype data at each locus. Bars represent allele frequencies, and error bars indicate 95% confidence intervals. The figure displays the most frequent alleles at each locus to facilitate comparison of HLA variation distributions across clinical groups; alleles not shown are included in the complete allele-level frequency dataset in **Supplementary Table 4**.

Per-locus allele frequencies, stratified by diagnostic group across all nine HLA loci, are presented in **Supplementary Table 4** and summarised graphically in **Figure 1**. Across the study population, DPA101:03:01 was the most frequent allele, with frequencies ranging from 62.5% in Healthy controls to 76.5% in ICU patients, 72.4% in Moderate cases, 72.7% in Recovered participants, and 68.5% in Severe cases. Thus, although DPA101:03:01 was consistently common across all groups, its frequency varied moderately by clinical category rather than being uniformly distributed. Several other alleles showed more pronounced differences in frequency across diagnostic groups. In particular, HLA-C06:02:01 increased from 11.7% in Healthy controls to 17.7% in ICU, 17.3% in Recovered, and 21.8% in Severe cases, while HLA-DQA102:01:01 increased from 12.5% in Healthy controls to 20.6% in ICU, 22.7% in Recovered, and 22.6% in Severe cases. These descriptive frequency gradients are consistent with potential disease-associated differences but should not be interpreted as evidence of association in isolation, as they do not account for covariates or multiple testing. The overlapping confidence intervals across several diagnostic strata, together with the relatively small size of individual subgroups, further warrant cautious interpretation; formal covariate-adjusted association analyses are therefore presented separately below.

Covariate-adjusted, per-locus HIBAG association testing (Severity ~ HLA + Age + Sex + Blood Group; dominant model) identified 22 HLA alleles that reached nominal significance (P < 0.10) for severe/critical COVID-19 in the primary model (Non-Severe Reference = Moderate + Recovered + Healthy; n = 364; **Table 4**). The strongest and most precisely estimated associations were HLA-C*06:02:01 (OR 2.50, P = 0.001) and HLA-DQB1*02:02:01 (OR 2.25, P = 0.003), both with comparatively narrow confidence intervals and the lowest FDR-adjusted q-values in the dataset (q = 0.36 and 0.48, respectively). Several additional alleles reached nominal significance but had markedly wide confidence intervals, reflecting low allele counts in this sample (HLA-A*29:02:01, OR 14.11, 95% CI 1.43–138.81; HLA-B*13:02:01, OR 12.78, 95% CI 1.31–124.18; HLA-DPA1*02:01:08, OR 6.40, 95% CI 1.13–36.26; HLA-B*14:01:01, OR 8.59, 95% CI 0.67–109.78); these estimates warrant particular caution. One allele, HLA-A*31:01:02, showed a nominally significant protective association (OR 0.26, 95% CI 0.08–0.83, P = 0.023), and two additional alleles trended protective without reaching P < 0.05 (HLA-DQB1*03:01:01, P = 0.064; HLA-DRB1*11:01:01, P = 0.066). No allele retained significance after Benjamini–Hochberg correction (FDR-adjusted q ranging from 0.36 to 0.99), consistent with the modest analytic sample size available for HLA association testing (n = 364, of the full 494-participant clinical cohort) and reduced power within the ICU stratum (n = 36).

**Table 4:** HLA alleles nominally associated with COVID-19 severity.

Allele	OR (95% CI)	p-value	FDR	Effect
C*06:02:01	2.50 [1.43, 4.36]	0.001295722	0.3615065	Risk
DQB1*02:02:01	2.25 [1.31, 3.88]	0.003474425	0.4846823	Risk
DRB1*15:03:01	4.65 [1.38, 15.67]	0.013013095	0.9930684	Risk
B*50:01:01	2.04 [1.11, 3.76]	0.022372404	0.9930684	Risk
A*31:01:02	0.26 [0.08, 0.83]	0.022787986	0.9930684	Protective
A*29:02:01	14.11 [1.43, 138.81]	0.023236065	0.9930684	Risk
B*13:02:01	12.78 [1.31, 124.18]	0.028108295	0.9930684	Risk
DPA1*02:01:08	6.40 [1.13, 36.26]	0.035817803	0.9930684	Risk
DQA1*01:01:02	2.65 [1.06, 6.61]	0.036601056	0.9930684	Risk
DRB1*01:02:01	2.65 [1.06, 6.61]	0.036601056	0.9930684	Risk
DRB4*01:03:01	1.70 [1.01, 2.87]	0.045047760	0.9930684	Risk
DQA1*02:01:01	1.71 [1.00, 2.92]	0.050643866	0.9930684	Risk
DQB1*03:01:01	0.53 [0.27, 1.04]	0.063656055	0.9930684	Protective
DRB1*11:01:01	0.14 [0.02, 1.14]	0.065832727	0.9930684	Protective
DPB1*105:01:01	3.95 [0.90, 17.32]	0.068249305	0.9930684	Risk
A*30:01:01	2.64 [0.90, 7.78]	0.077645794	0.9930684	Risk
B*42:01:01	5.97 [0.82, 43.71]	0.078395830	0.9930684	Risk
DRB1*07:01:01	1.60 [0.94, 2.71]	0.083403937	0.9930684	Risk
A*33:01:01	3.10 [0.85, 11.35]	0.086963894	0.9930684	Risk
DQB1*04:02:01	2.72 [0.86, 8.60]	0.087642817	0.9930684	Risk
C*14:02:01	2.32 [0.87, 6.18]	0.093914501	0.9930684	Risk
B*14:01:01	8.59 [0.67, 109.78]	0.098061405	0.9930684	Risk

Alleles with P < 0.10 for association with severe/critical-ICU disease versus the non-severe reference group (Moderate, Recovered, Healthy; n = 364), from per-locus, covariate-adjusted logistic regression (dominant genetic model; adjusted for age, sex, and ABO blood group). Abbreviations: OR, odds ratio; CI, confidence interval; FDR, Benjamini–Hochberg-corrected p-value. No allele survived FDR correction (q range: 0.36–0.99); several associations with markedly wide confidence intervals (e.g., HLA-A*29:02:01, HLA-B*13:02:01, HLA-B*14:01:01) reflect low allele counts in this sample and should be interpreted with particular caution. All findings are hypothesis-generating replication candidates, not validated genetic determinants.

## 4 Discussion

This study, which included COVID-19 patients with varying disease severity and healthy controls, showed trends that aligned with global findings on the immunogenetic determinants of SARS-CoV-2 outcomes. These findings suggest that specific HLA alleles may modify individual susceptibility to severe COVID-19. Alleles such as HLA-C*06:02:01, HLA-B*13:02:01, and HLA-DRB1*15:03:01 were associated with increased odds of severe or critical/ICU-level disease, whereas HLA-A*31:01:02 showed a protective effect. These results are broadly consistent with prior work implicating HLA polymorphisms in T-cell responses by influencing the duration and immunostimulatory potential of viral peptide presentation (10, 11). HLA genetics is thought to shape COVID-19 severity largely through differences in SARS-CoV-2 antigen presentation, which in turn determine the strength and specificity of the individual immune response (16). Large global studies have identified male sex as a risk factor for COVID-19 hospitalisation and mortality (17, 18); In our cohort, however, no statistically significant sex association was observed (OR 1.26, 95% CI 0.83–1.92, P = 0.289; **Table 2C**). This discrepancy may partly reflect the limited statistical power of our sample after stratification, though the near-identical hospitalisation rates between females and males (64% vs. 64%) may also reflect recruitment at a single tertiary referral centre during peak pandemic surges, when triage-driven admission criteria could have minimised sex-based differences that might otherwise emerge in more heterogeneous settings. Larger, population-representative Gulf Cooperation Council (GCC) cohorts are needed to conclusively characterise the role of sex in COVID-19 severity within this population. Reassuringly, the most prevalent alleles in our cohort overlap substantially with those reported in the neighbouring Saudi population — including HLA-C*06:02, HLA-B*50:01, and HLA-DRB1*07:01 (19)—supporting the regional plausibility of our allele-frequency estimates.

Antigen presentation to CD8^1^ and CD4^1^ T cells is governed by HLA molecules, shaping immune memory and viral clearance (9). During the 2003 SARS outbreak, HLA-B*46:01 was associated with more severe SARS-CoV infection, implicating host immunogenetic variation in outcome (12), while in silico modelling has suggested that HLA-B*15:03 may bind a broader range of peptides and confer cross-protective immunity (13). In our cohort, the enrichment of HLA-C*06:02:01 and HLA-B*13:02:01 among severe/critical cases is consistent with the hypothesis that inefficient peptide presentation — impairing antiviral cytotoxic T-cell responses — may predispose to heightened inflammation and prolonged viral persistence, though this remains a plausible mechanistic interpretation rather than a demonstrated one in this dataset.

Consistent with Liu et al. (2020), age emerged as the dominant clinical risk factor for severe COVID-19 in our cohort, with older participants at substantially greater risk of ICU admission (2). This mirrors observations from other cohorts worldwide in which sex, unlike age, showed no consistent independent association with COVID-19 severity (20).

The HLA-DPA1*01:03:01 allele showed a consistently high within-locus frequency across all diagnostic strata (62.5–74.2%; **Supplementary Table 4**), suggesting it is a dominant, fixed feature of the DPA1 repertoire in the Kuwait-based population rather than evidence of broader multi-locus haplotype stability; confirming the latter would require haplotype-level analysis across linked loci in an independent Kuwait-Residentreference cohort (15). Non-genetic factors may also confound HLA–COVID-19 associations: a large cohort study found that, after adjusting for exposure risk and socioeconomic status, only a limited number of HLA alleles remained significantly associated with SARS-CoV-2 infection (21), underscoring the need for similarly rigorous adjustment for confounders in future regional studies.

### 4.1 Statistical power and unmeasured confounding

The lack of FDR-significant HLA-severity association here should be seen as due to limited power and unmeasured covariates, not necessarily the absence of a true effect. We estimated the minimum detectable odds ratio (MDOR) at 80% power (α=0.05) for allele carrier frequencies in the Severe/Critical-ICU (n=102) vs. Non-Severe group (n=262; primary model; **Supplementary Table 7**). Detecting a 1%-frequency allele needed an OR of about 10, and a 2%-frequency allele about 6; with multiple-testing correction (effective α ≈ 1.79×10^-4^ across 279 alleles), thresholds rose to 23 and 13. Some alleles with nominal associations, like HLA-DRB1*15:03:01, HLA-A*29:02:01, and HLA-B*13:02:01, are low frequency and low power, so our study might not detect effects of this size at the corrected significance level even if they are real.

Several important covariates in COVID-19 severity analyses, like comorbidities, body mass index, and vaccination status, were unavailable in this dataset. Comorbidities and BMI, key predictors of severity, were not recorded. Vaccination status, which overlaps with Kuwait’s vaccine rollout, was not documented and could not be adjusted for; vaccinated individuals tend to be less severe, and vaccination correlates with age and time. Healthy controls were required to be seronegative on the anti-spike antibody assay, likely enriching them for unvaccinated individuals, while patient vaccination status was unknown, creating systematic group differences. Also, data on calendar period, circulating variants, and treatment received were missing, despite these factors influencing severity. These gaps mean the HLA associations cannot be fully distinguished from known severity predictors, and any allele-severity link might reflect confounding factors rather than true genetic effects.

Limitations. This study is among the few HLA investigations of COVID-19 severity in Kuwaiti residents and, to our knowledge, represents the first immunogenetic characterisation of Kuwait-based COVID-19 patients at this resolution, but several limitations warrant caution in interpreting its findings. The modest sample size, particularly after stratification into five severity categories, limited statistical power and produced wide confidence intervals for low-frequency alleles; this likely explains why several HLA-severity associations that were nominally significant did not survive FDR correction (adjusted q ranging from 0.36 to 0.99, with HLA-C*06:02:01 and HLA-DQB1*02:02:01 closest to, though still short of, conventional significance). Hospital-based enrolment during the pandemic may have introduced additional selection bias, compounded by uneven dropout between the ICU and other severity groups in the genotype-linked data. A further limitation is that nationality and ancestry data were not collected at enrolment: Kuwait’s population includes Arab, South Asian, and other ancestries with differing baseline HLA allele frequencies, and expatriate and citizen subpopulations also differ systematically in age, occupation, and healthcare access, any of which could confound the reported associations — particularly for low-frequency alleles such as HLA-DRB1*15:03:01, more common in populations of African ancestry. Because ancestry could not be adjusted for, all associations reported here should be interpreted with this potential confounding in mind, and future studies should collect self-reported nationality and ancestry data at enrolment to disentangle genetic confounding from true disease-severity relationships. Finally, while statistical association alone cannot determine mechanism, functional validation via HLA antigen-presentation assays and T-cell receptor profiling is necessary to confirm the biological significance of any candidate allele. Overall, since no HLA-severity associations in this study remained significant after correcting for multiple testing, these findings should be viewed as hypothesis-generating observations that need replication in larger, independent, ancestry-adjusted cohorts before they can be used in translational applications — such as risk stratification or targeted clinical treatments. Nonetheless, the allele-frequency landscape offers a reference for the Kuwait-based population, which may guide future, well-powered immunogenetic studies of COVID-19 and other viral diseases in the region, highlighting the important role of regional genomic research in improving disease models for coronaviruses and emerging pathogens.

## 5 Conclusion

Despite rising global interest in host immunogenetics amid viral diseases like COVID-19 and SARS, little research has focused on Middle Eastern and Arab populations. This study helps fill that gap by providing one of the first detailed analyses of HLA allele frequencies and their association with COVID-19 outcomes in Kuwait. Age was a stronger predictor of severity than HLA genotype. While some alleles (e.g., HLA-C*06:02, HLA-DRB1*15:03) showed nominal links to severe disease, none were significant after FDR correction. This null result mainly reflects limited statistical power and unmeasured confounders (comorbidities, vaccination, ancestry) rather than a true absence of effect. These associations are hypotheses needing replication and validation, not for clinical use. The allele-frequency data serve as a reference for future studies on COVID-19 and viral diseases in Kuwait.

## Data Availability

The original contributions presented in the study are included in the article/**Supplementary Material**. Further inquiries can be directed to the corresponding author.

## Supplementary material

Supplementary Table 1 Clinical case definitions used to classify COVID-19 disease severity in this study, adapted from the U.S. Food and Drug Administration guidance document COVID-19: Developing Drugs and Biological Products for Treatment or Prevention: Guidance for Industry (February 2021). This FDA scheme is the sole severity classification applied throughout the manuscript; the thresholds shown here (Moderate: RR ≥ 20/min, SpO_2_ > 93% on room air, HR ≥ 90/min; Severe: RR ≥ 30/min, HR ≥ 125/min, SpO_2_ ≤ 93% on room air, or PaO_2_/FiO_2_ < 300; Critical/ICU: respiratory failure requiring advanced respiratory support, shock, or multi-organ dysfunction/failure) correspond exactly to those cited in §2.2. Definitions are provided for five FDA-defined categories: Asymptomatic Infection, Mild, Moderate, Severe, and Critical COVID-19. No participants in this cohort met the criteria for Asymptomatic Infection. The Mild category (referred to as “Minor” during initial data entry) was harmonised with the Moderate group prior to analysis. The Recovered stratum analysed elsewhere in this study is not an FDA acute-severity category; it is defined independently by time since infection (enrolment within 90 days of confirmed infection) and symptom resolution, and is not covered by the definitions in this table.

Supplementary Table 2 HLA genotyping call rates by locus and diagnostic group. Number and percentage of participants with complete genotype calls at each of the nine HLA loci [**(A–C)**, DPA1, DPB1, DQA1, DQB1, DRB1, DRB3/4/5], stratified by diagnostic group (Healthy, Recovered, Moderate, Severe, ICU). Locus totals reflect the number of participants with complete clinical and genetic data available in each group (Healthy n = 60, Recovered n = 75, Moderate n = 125, Severe n = 62, ICU n = 34; total n = 356). Call rates were 100% across all loci and diagnostic groups, indicating no missing genotype calls among participants with complete clinical/covariate data.

Supplementary Table 3 Association between genotyping missingness and clinical outcome, by locus. P-values from tests of association between HLA genotyping missingness and diagnostic outcome at each of the nine loci. As call completeness was 100% at every locus across all diagnostic groups (**Supplementary Table 2**), the identical p-value obtained at each locus reflects the fixed group-size distribution rather than a locus-specific missingness effect, confirming that genotyping completeness did not differ by disease severity.

Supplementary Table 4 Allele frequencies at nine HLA loci by diagnostic group. Frequency (count, percentage, and 95% confidence interval) of each observed allele at the nine genotyped HLA loci **(A–C)** (DPA1, DPB1, DQA1, DQB1, DRB1, DRB3/4/5), stratified by diagnostic group (Healthy, Recovered, Moderate, Severe, ICU). Frequencies were calculated using a locus-specific 2N denominator (twice the number of participants genotyped at each locus within each group). These data underlie the summary distributions presented in **Figure 1**.

Supplementary Table 5 Full HLA association test results across all loci. Complete results of covariate-adjusted logistic regression association tests (severity ~ HLA + age + sex + blood group; dominant genetic model) for all HLA alleles meeting the 1% phenotypic frequency threshold across the nine genotyped loci, comparing the Severe/Critical-ICU group with the non-severe reference group (Moderate, Recovered, Healthy). Odds ratios (OR), 95% confidence intervals, unadjusted p-values, and Benjamini–Hochberg FDR-corrected q-values are reported for every tested allele; the nominally significant subset (P < 0.05) is presented in the main-text Table 5.

Supplementary Table 6 **(A)** HIBAG-based HLA association results from sensitivity analysis using an alternative reference population. HLA allele associations were re-evaluated using imputed HLA genotypes via the HIBAG algorithm after excluding healthy controls to test the robustness of primary findings. For each allele, the table shows the odds ratio (OR), 95% confidence interval (CI), P values, FDR, and Bonferroni adjustments, with ORs >1 indicating increased disease risk and <1 indicating protection. The same analysis framework was used for sensitivity analysis to check if associations remained consistent with a restricted control group. Significance levels account for multiple testing across alleles. **(B)**. Concordance of HLA allele associations between the primary and sensitivity analyses. Comparison of HLA association results from the primary and sensitivity analyses using an alternative reference population. For each HLA allele, the table shows effect estimates and significance measures from both analyses, including odds ratios (ORs), nominal P values, and FDR-adjusted P values. It also summarizes the direction and significance of associations across the approaches, allowing assessment of reproducibility and robustness. Concordant associations have consistent effect directions and similar statistical evidence, while discordant findings show changes in effect or significance after modifying the reference population. This comparison assesses the stability of HLA associations and helps identify robust disease-associated alleles from those influenced by control group composition.

Supplementary Table 7 Statistical power to detect minimum detectable odds ratios (MDORs) across allele carrier frequencies. Estimated MDORs are presented for the primary analysis (n=102 vs. 262) and sensitivity analysis (n=102 vs. 202) across a range of allele carrier frequencies (1–30%). The final column presents MDORs under the FDR-equivalent significance threshold (α ≈ 1.79 × 10^-4^) for the primary analysis.
